# Comparative Genomics of Listeria monocytogenes Isolates from Ruminant Listeriosis Cases in the Midwest United States

**DOI:** 10.1128/spectrum.01579-22

**Published:** 2022-10-31

**Authors:** Maria X. Cardenas-Alvarez, Hui Zeng, Brett T. Webb, Rinosh Mani, Marina Muñoz, Teresa M. Bergholz

**Affiliations:** a Department of Pharmacology, University of North Carolina, Chapel Hill, North Carolina, USA; b Department of Food Science and Human Nutrition, Michigan State University, East Lansing, Michigan, USA; c Veterinary Diagnostic Laboratory, North Dakota State University, Fargo, North Dakota, USA; d Veterinary Diagnostic Laboratory, Michigan State University, Lansing, Michigan, USA; e Centro de Investigaciones en Microbiología y Biotecnología-UR (CIMBIUR), Facultad de Ciencias Naturales, Universidad del Rosario, Bogotá, Colombia; Agriculture and Agriculture-Food Canada

**Keywords:** ruminant, listeriosis, neurologic, fetal infection, lineage, clonal complex, LIPI-1, internalins, LIPI-3, LIPI-4

## Abstract

Ruminants are a well-known reservoir for Listeria monocytogenes. In addition to asymptomatic carriage of the pathogen, ruminants can also acquire listeriosis and develop clinical manifestations in the form of neurologic or fetal infections, similar to those occurring in humans. Genomic characterization of ruminant listeriosis cases in Europe have identified lineage 1 and 2 strains associated with infection, as well as clonal complexes (CCs) that are commonly isolated from human cases of listeriosis; however, there is little information on the diversity of L. monocytogenes from ruminant listeriosis in the United States. In this study, we characterized and compared 73 L. monocytogenes isolates from ruminant listeriosis cases from the Midwest and the Upper Great Plains collected from 2015 to 2020. Using whole-genome sequence data, we classified the isolates and identified key virulence factors, stress-associated genes, and mobile genetic elements within our data set. Our isolates belonged to three different lineages: 31% to lineage 1, 53% to lineage 2, and 15% to lineage 3. Lineage 1 and 3 isolates were associated with neurologic infections, while lineage 2 showed a greater frequency of fetal infections. Additionally, the presence of mobile elements, virulence-associated genes, and stress and antimicrobial resistance genes was evaluated. These genetic elements are responsible for most of the subgroup-specific features and may play a key role in the spread of hypervirulent clones, including the spread of hypervirulent CC1 clone commonly associated with disease in humans, and may explain the increased frequency of certain clones in the area.

**IMPORTANCE** Listeria monocytogenes affects humans and animals, causing encephalitis, septicemia, and abortions, among other clinical outcomes. Ruminants such as cattle, goats, and sheep are the main carriers contributing to the maintenance and dispersal of this pathogen in the farm environment. Contamination of food products from farms is of concern not only because many L. monocytogenes genotypes found there are associated with human listeriosis but also as a cause of significant economic losses when livestock and food products are affected. Ruminant listeriosis has been characterized extensively in Europe; however, there is limited information about the genetic diversity of these cases in the United States. Identification of subgroups with a greater ability to spread may facilitate surveillance and management of listeriosis and contribute to a better understanding of the genome diversity of this pathogen, providing insights into the molecular epidemiology of ruminant listeriosis in the region.

## INTRODUCTION

Listeria monocytogenes is a foodborne pathogen of concern for humans and ruminants. In humans, listeriosis can lead to central nervous system infections, septicemia, and neonatal infections in pregnant women (1). Similar clinical manifestations, as well as mastitis and eye infections, can occur in ruminants (2, 3). Healthy ruminants can also carry L. monocytogenes in their gastrointestinal tract and are considered a major reservoir of the pathogen; they shed L. monocytogenes into the environment, with the potential to enter the food supply. Fecal shedding can occur among a significant portion of animals in a herd, with reports of 4% to 46% of cattle and 14% of sheep with L. monocytogenes detected in fecal samples (4–6).

L. monocytogenes is a genetically diverse species and strains can be classified into phylogenetic lineages, clonal complexes (CCs), and sequence types (STs) using 7-gene multilocus sequence typing (MLST) (7), as well as into lineages, sublineages (SLs), and cgMLST types (CTs) using whole-genome sequencing data (8), which extends the MLST concept to a larger number of genes in the core genome. All L. monocytogenes strains possess the key virulence genes responsible for invasion of and multiplication in host cells, present on *Listeria* pathogenicity island 1 (LIPI-1) (1). Phenotypes associated with hypervirulence have been linked to accessory genome components, including *Listeria* pathogenicity islands 3 (LIPI-3) and 4 (LIPI-4), and internalin genes (9). Multiple assessments of L. monocytogenes subtypes have identified frequently isolated CCs, and certain subgroups have been associated with specific virulence phenotypes. While strains of CC1, CC4, and CC6 are considered hypervirulent and are associated with severe disease in humans (10), CC1 strains are also associated with severe disease (rhombencephalitis) in ruminants (11, 12).

In a survey of 187 ruminant rhombencephalitis-associated L. monocytogenes strains from the United Kingdom and Switzerland, Dreyer and colleagues reported that the majority of isolates belonged to CC1, CC4, and CC412 (11). Papic and colleagues examined a set of 350 L. monocytogenes isolates from cases of ruminant listeriosis in four countries across Europe, comparing those from cases of rhombencephalitis and from maternal-neonatal infections. Isolates of CC1 were significantly associated with rhombencephalitis, while those from CC37 and CC6 were significantly associated with abortion (12). They also assessed diversity of L. monocytogenes isolates from natural environments, including on farms, and found that while CC1 strains were frequently isolated from clinical samples, strains from CC9, CC14, and CC29 were significantly associated with environmental samples (12).

Surveys of L. monocytogenes on ruminant farms have described the diversity and most commonly isolated subtypes. Castro and colleagues identified persistent clones of L. monocytogenes that were repeatedly isolated from environmental samples on dairy farms in Finland. These included isolates of ST20 (CC20), ST14 (CC14), ST91 (CC14), and ST37 (CC37), and the overwhelming majority of isolates were from lineage 2 (13). Subtyping of L. monocytogenes isolates from food products in France and other countries in Europe revealed that strains of CC1, CC2, CC37, and CC101 were common in dairy products (14, 15). Palacios-Gorba and colleagues conducted a longitudinal study of 19 farms in Spain, isolating L. monocytogenes from ruminant fecal and farm environmental samples; where they found that 70% of L. monocytogenes isolates were from lineage 1, 30% were from lineage 2, and the most prevalent SLs were SL1 (CC1), SL219 (CC4), SL26 (CC26), and SL87 (CC87) (6). Taken together, these studies demonstrate that L. monocytogenes clones responsible for significant human disease are found in ruminants, in the ruminant farm environment, and in milk and other dairy products.

While diversity of L. monocytogenes isolates from ruminant listeriosis has been characterized extensively in Europe, few studies have examined isolates from cases of ruminant listeriosis in the United States. Previous work by Steckler and colleagues used MLST to compare L. monocytogenes isolates from ruminant listeriosis in two regions of the United States and found that lineage 2 isolates were more frequent than lineage 1 isolates. Some STs were common between the two regions, such as ST7 and ST191, while others were unique to each region (16). In this study, we built on our previous collection of ruminant listeriosis isolates and used whole-genome sequence data to classify and compare 73 isolates from neurologic and fetal infections in ruminants. We also identified key virulence factors and stress and antimicrobial resistance (AMR)-associated genes along with mobile genetic elements to study the genetic diversity of L. monocytogenes isolates causing ruminant listeriosis in the Midwest and the Upper Great Plains.

## RESULTS

### Diversity of ruminant listeriosis isolates.

**FIG 1:**
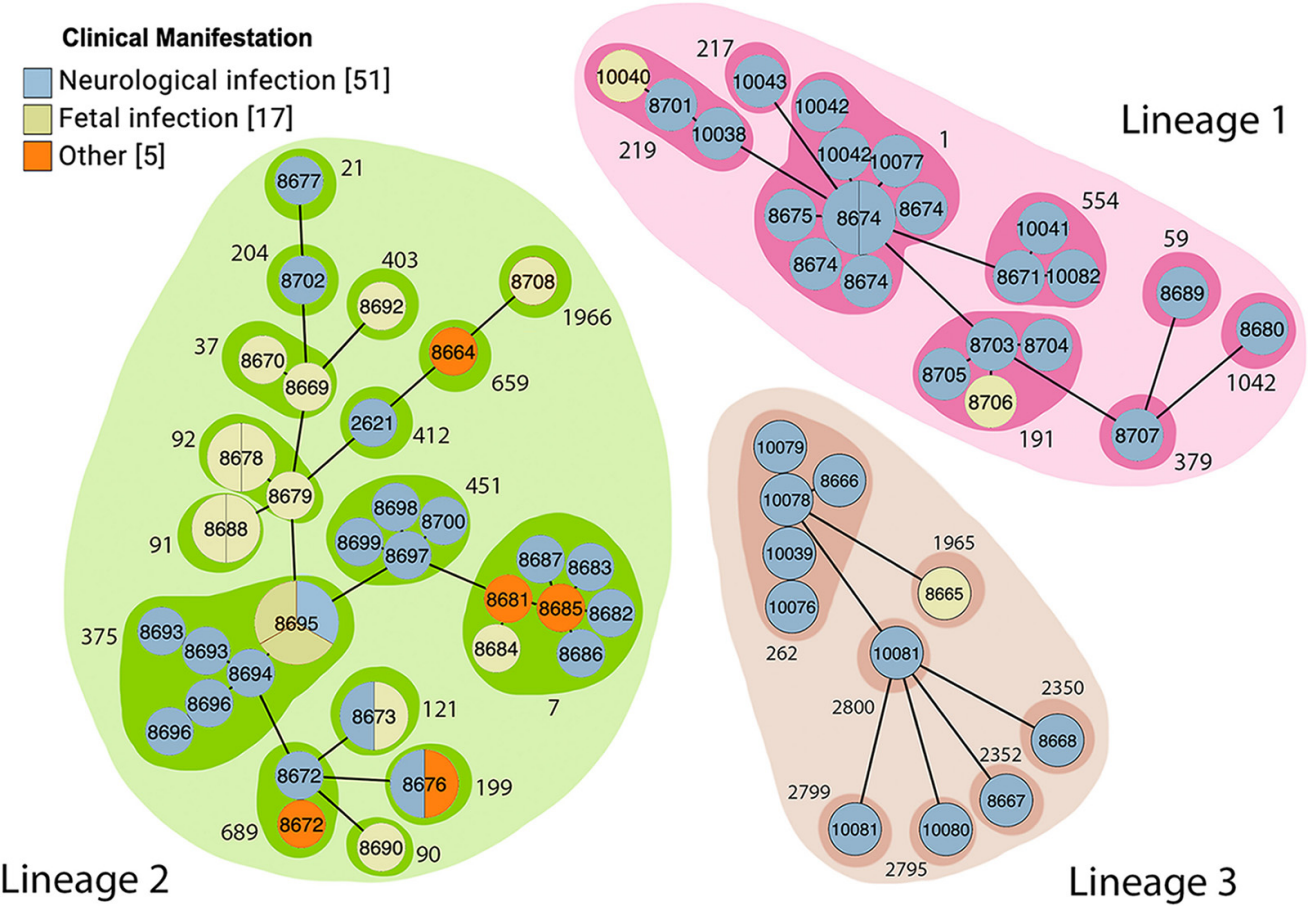
Minimum spanning tree based on cgMLST profiles constructed using GrapeTree. cgMLST types (CTs) are shown inside each circle. CTs that belong to the same sublineage (SLs) are grouped with a shaded area labeled with the corresponding SL (outside number). The size of each circle represents the number of isolates within each CT. Different circle colors represent the clinical manifestations observed in the ruminant from which each strain was isolated.

Most isolates were collected from cattle (64.4% [47/73]), with the remaining 23.3% from sheep (17/73) and 12.3% from goats (9/73). We used cgMLST to classify the isolates. Among the 73 isolates, 23 (31.5%) belonged to lineage 1, 39 (53.4%) belonged to lineage 2, and 11 (15.1%) belonged to lineage 3 (Table 1). The isolates belonged to 31 SLs, with 8 different SLs in lineage 1, 16 different SLs in lineage 2, and 7 different SLs in lineage 3. SL1 (9/73 [12.3% of the total isolates]) was most frequent among lineage 1 isolates, followed by SL375 (8/73 [11.0%]). SL7 (7/73 [9.6%]) was the most frequent among lineage 2 isolates, and SL262 (5/73 [6.8%]) was the most frequent among lineage 3. Other prevalent SLs included SL191 (4/73 [5.5%]), SL219 (3/73 [4.1%]), and SL554 (3/73 [4.1%]) in lineage 1 and SL451 (4/73 [5.5%]) in lineage 2. Among the 73 isolates, 58 CTs were identified, and all of them were new CTs and were added to the Institut Pasteur MLST database. Among the 58 cgMLST types, 47 (81%) unique CTs were found only for a single isolate, and 11 (19%) CTs shared from two to five allelic similarities (Fig. 1). Isolates with the same CT were from the same geographic location, although the year of isolation and source of the isolate differed. For example, among five CT8674 strains isolated in North Dakota, one was isolated in 2015 from a bovine brain, while four were isolated in 2017 from sheep brain samples.

**TABLE 1:** L. monocytogenes isolated from cases of ruminant listeriosis used in this study

Lineage	Sublineage	cgMLST type	Isolate ID	Source	Clinical manifestation	Yr isolated	State	Serotype	Accession no.^*a*^
1	SL1	CT8674	TB0359	Bovine	Neurologic	2015	ND	IVb	SRR17426669, SRR17430284
	SL1	CT8674	TB0509	Ovine	Neurologic	2017	ND	IVb	SRR6116335, SRR17681511
	SL1	CT8674	TB0510	Ovine	Neurologic	2017	ND	IVb	SRR6116322, SRR17681510
	SL1	CT8674	TB0513	Ovine	Neurologic	2017	ND	IVb	SRR6116308, SRR17681523
	SL1	CT8674	TB0514	Ovine	Neurologic	2017	ND	IVb	SRR6116057, SRR17682119
	SL1	CT8675	TB0654	Bovine	Neurologic	2018	SD	IVb	SRR17681174
	SL1	CT10042	TB0703	Ovine	Neurologic	2019	MI	IVb	SRR15498165
	SL1	CT10042	TB0705	Ovine	Neurologic	2019	MI	IVb	SRR16134512
	SL1	CT10077	TB0707	Ovine	Neurologic	2020	MI	IVb	SRR16133661
	SL59	CT8689	TB0656	Bovine	Neurologic	2019	MN	IIb	SRR17681170
	SL191	CT8703	TB0405	Ovine	Neurologic	2015	ND	IIb	SRR17621672
	SL191	CT8704	TB0404	Ovine	Neurologic	2015	ND	IIb	SRR17621673
	SL191	CT8705	TB0695	Bovine	Neurologic	2020	ND	IIb	SRR12120241
	SL191	CT8706	TB0693	Bovine	Fetal infection	2020	ND	IIb	SRR12120243
	SL217	CT10043	TB0704	Caprine	Neurologic	2019	MI	IVb	SRR15498160
	SL219	CT8701	TB0621	Bovine	Neurologic	2018	MN	IVb	SRR7690607
	SL219	CT10038	TB0699	Bovine	Neurologic	2016	MI	IVb	SRR15498164
	SL219	CT10040	TB0701	Ovine	Fetal infection	2019	MI	IVb	SRR15498158
	SL379	CT8707	TB0579	Bovine	Neurologic	2017	ND	IIb	SRR17578412, SRR17681518
	SL554	CT8671	TB0364	Bovine	Neurologic	2015	MN	IVb-v1	SRR17621674
	SL554	CT10041	TB0702	Ovine	Neurologic	2019	MI	IVb-v1	SRR15498163
	SL554	CT10082	TB0697	Caprine	Neurologic	2020	ND	IVb-v1	SRR16134482
	SL1042	CT8680	TB0657	Caprine	Neurologic	2019	ND	IIb	SRR17681169
2	SL7	CT8687	TB0358	Bovine	Neurologic	2015	ND	IIa	SRR17426670, SRR17430285
	SL7	CT8683	TB0360	Bovine	Neurologic	2015	ND	IIa	SRR17426668, SRR17430283
	SL7	CT8681	TB0488	Bovine	Healthy	2016	ND	IIa	SRR17621676, SRR17681513
	SL7	CT8682	TB0451	Bovine	Neurologic	2016	ND	IIa	SRR6116309, SRR17681517
	SL7	CT8684	TB0684	Bovine	Fetal infection	2019	ND	IIa	SRR17681163
	SL7	CT8685	TB0452	Bovine	Other	2016	ND	IIa	SRR17621669, SRR17681516
	SL7	CT8686	TB0486	Caprine	Neurologic	2016	ND	IIa	SRR17621677, SRR17681514
	SL21	CT8677	TB0363	Ovine	Neurologic	2015	MN	IIa	SRR17621675
	SL37	CT8669	TB0632	Bovine	Fetal infection	2018	ND	IIa	SRR7690615
	SL37	CT8670	TB0522	Bovine	Fetal infection	2017	ND	IIa	SRR6113306, SRR17681522
	SL90	CT8690	TB0577	Bovine	Fetal infection	2017	ND	IIa	SRR17578411, SRR17681519
	SL91	CT8688	TB0353	Bovine	Fetal infection	2015	SD	IIa	SRR17426674, SRR17430282
	SL91	CT8688	TB0354	Bovine	Fetal infection	2015	SD	IIa	SRR17426673, SRR17430286
	SL92	CT8678	TB0407	Bovine	Fetal infection	2016	ND	IIa	SRR17621671, SRR17681527
	SL92	CT8678	TB0408	Bovine	Fetal infection	2016	ND	IIa	SRR17621670, SRR17681526
	SL92	CT8679	TB0527	Bovine	Fetal infection	2017	ND	IIa	SRR6113191, SRR17681521
	SL121	CT8673	TB0511	Bovine	Fetal infection	2017	ND	IIa	SRR6116326, SRR17681525
	SL121	CT8673	TB0512	Bovine	Neurologic	2017	ND	IIa	SRR6116310, SRR17681524
	SL199	CT8676	TB0361	Bovine	Other	2015	ND	IIa	SRR17621681
	SL199	CT8676	TB0362	Bovine	Neurologic	2015	ND	IIa	SRR17621680
	SL204	CT8702	TB0485	Caprine	Neurologic	2016	WY	IIa	SRR17621678
	SL375	CT8693	TB0687	Bovine	Neurologic	2020	ND	IIa	SRR17681172
	SL375	CT8693	TB0688	Bovine	Neurologic	2020	ND	IIa	SRR17681171
	SL375	CT8694	TB0696	Bovine	Neurologic	2020	ND	IIa	SRR12120240
	SL375	CT8695	TB0689	Bovine	Fetal infection	2020	ND	IIa	SRR12120247
	SL375	CT8695	TB0690	Bovine	Neurologic	2020	ND	IIa	SRR12120246
	SL375	CT8695	TB0691	Bovine	Fetal infection	2020	ND	IIa	SRR12120245
	SL375	CT8696	TB0663	Bovine	Neurologic	2019	ND	IIa	SRR17681167
	SL375	CT8696	TB0683	Bovine	Neurologic	2019	ND	IIa	SRR17681164
	SL403	CT8692	TB0630	Bovine	Fetal infection	2018	ND	IIa	SRR8502748
	SL412	CT2621	TB0659	Bovine	Neurologic	2019	ND	IIa	SRR17681168
	SL451	CT8697	TB0571	Bovine	Neurologic	2017	ND	IIa	SRR6113911, SRR17681520
	SL451	CT8698	TB0356	Bovine	Neurologic	2015	ND	IIa	SRR17426672
	SL451	CT8699	TB0677	Caprine	Neurologic	2019	ND	IIa	SRR17681166
	SL451	CT8700	TB0481	Ovine	Neurologic	2016	ND	IIa	SRR17621679
	SL659	CT8664	TB0694	Ovine	Other	2020	ND	IIa	SRR12120242
	SL689	CT8672	TB0655	Ovine	Neurologic	2018	ND	IIa	SRR17681173
	SL689	CT8672	TB0678	Bovine	Other	2019	ND	IIa	SRR17681165
	SL1966	CT8708	TB0692	Ovine	Fetal infection	2020	ND	IIa	SRR12120244
3	SL262	CT8666	TB0453	Bovine	Neurologic	2016	SD	L	SRR17621668, SRR17681515
	SL262	CT10079	TB0673	Bovine	Neurologic	2015	NE	L	SRR16133811
	SL262	CT10080	TB0674	Bovine	Neurologic	2015	NE	L	SRR16134473
	SL262	CT10081	TB0675	Bovine	Neurologic	2015	NE	L	SRR16248686
	SL262	CT10081	TB0676	Bovine	Neurologic	2015	NE	L	SRR16248657
	SL1965	CT8665	TB0508	Ovine	Fetal infection	2017	ND	L	SRR6116311, SRR17681512
	SL2350	CT8668	TB0357	Bovine	Neurologic	2015	ND	L	SRR17426671
	SL2352	CT8667	TB0631	Bovine	Neurologic	2018	ND	L	SRR8502801
	SL2795	CT10039	TB0700	Caprine	Neurologic	2016	MI	L	SRR15498157
	SL2799	CT10076	TB0706	Caprine	Neurologic	2019	MI	L	SRR16133672
	SL2800	CT10078	TB0708	Caprine	Neurologic	2020	MI	L	SRR16134467

a When two accession numbers are present, they include both short- and long-read data sets.

Over the 5-year collection period, isolates from lineages 1 and 2 were identified from cases in multiple states. Lineage 3 isolates, which have rarely been reported in assessments of ruminant listeriosis, were isolated from cases in three different states over the 5-year period (Table 1). SL1, the most frequently isolated SL, was found in cases in three states over the course of the study. SL375 was isolated only from cases in North Dakota in 2019 and 2020, while SL7 was also isolated only from cases in North Dakota, but more frequently in 2015 and 2016.

### Association between clinical manifestations and lineage.

The majority of isolates were collected from ruminants with neurologic infections (51/73 [69.9%]), which was significantly higher (*P* < 0.05) than those collected from fetal clinical manifestations. To assess potential associations between clinical manifestations and lineage, we focused on those isolates from neurologic and fetal infections, as those classified as other infections were few and all belonged to lineage 2. In lineage 1, 91.3% (21/23) of isolates were associated with neurologic infections, and 8.7% (2/23) were associated with fetal infections, while lineage 3 had 90.9% (10/11) isolates associated with neurologic infections and 9.1% (1/11) isolates associated with fetal infections. Lineage 2 isolates were not as frequently associated with neurologic infections (20/39 [51.3%]) as were lineages 1 and 3 but did have a greater frequency of fetal infections (14/39 [35.6%]). Within all three lineages the frequency of clinical manifestations was significantly different (*P* < 0.05), with neurologic infections most frequent. In a comparison within the 3 clinical manifestations, the frequency of neurologic infections was found to be significantly lower in lineage 2 than in lineages 1 and 3, and the frequency of fetal infections was significantly higher in lineage 2 than in lineages 1 and 3.

### Virulence gene profiles.

**FIG 2:**
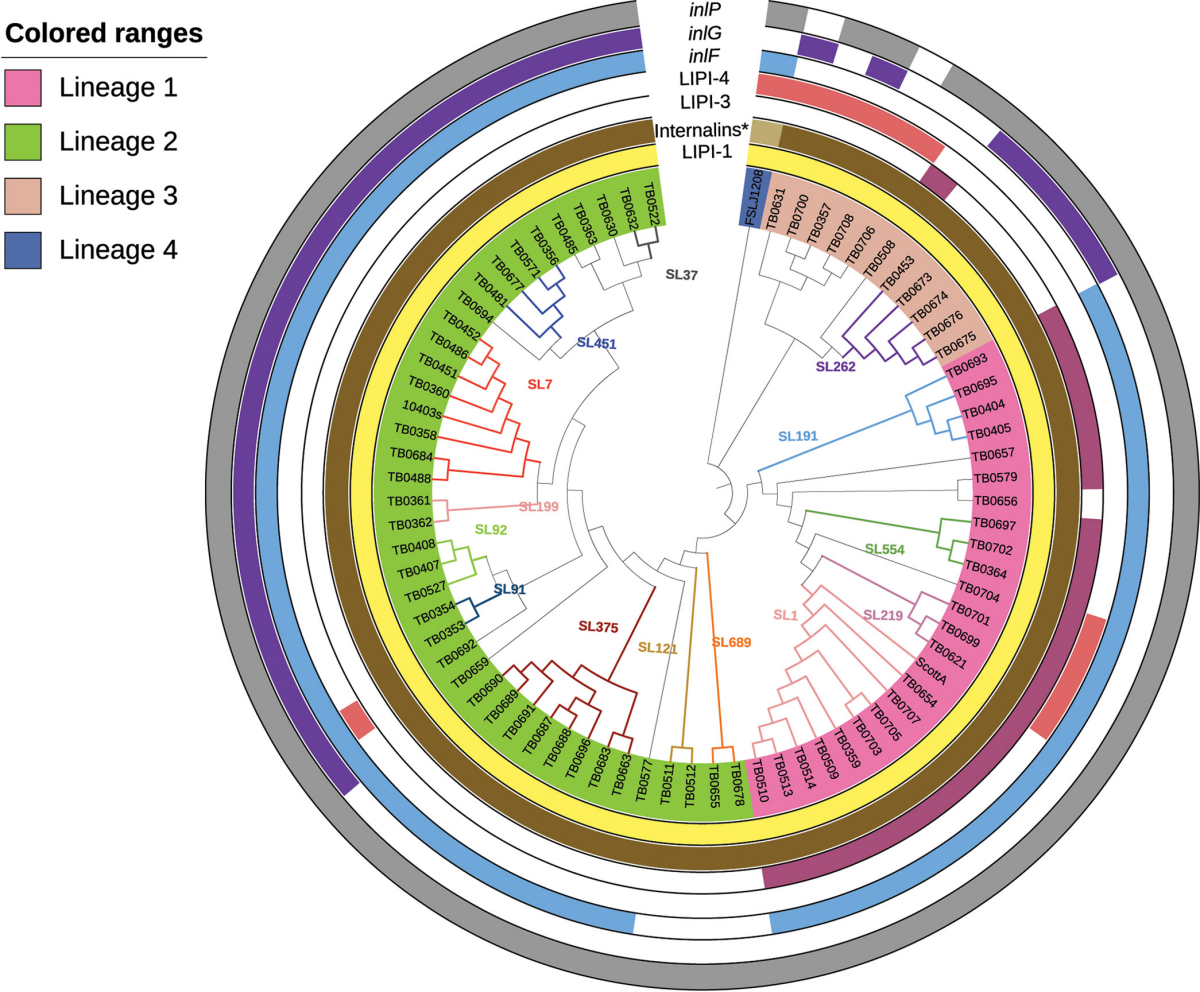
cgMLST-based phylogenetic tree of 73 isolates from ruminant listeriosis cases and three reference genomes. Each isolate identifier (ID) is colored by lineage, and sublineage branches are indicated on the dendrogram. Virulence factors LIPI-1, *inlF*, *inlG*, *inlP*, LIPI-3, and LIPI-4 are shown in the outer rings as color strips indicating presence/absence. The ring labeled “Internalins*” includes *inlA*, *inlB*, *inlC*, *inlE*, *inlH*, *inlJ*, and *inlK*. The light brown shade for reference strain FSLJ1208 indicates that it was missing genes *inlE*, *inlH*, and *inlJ*.

All isolates contained *Listeria* pathogenicity island 1 (LIPI-1; *prfA*, *plcA*, *plcB*, *actA*, *mpl*, and *hly*) (Fig. 2). We also assessed the presence of 10 internalin gene family members, including *inlAB*, -*C*, -*E*, -*F*, -*G*, -*H*, -*J*, -*K*, and -*P*. In our collection, 35.6% (26/73) of the isolates harbored all of the internalin genes screened, and all of them belonged to lineage 2. Likewise, 53.4% (39/73) carried nine and 11.0% (8/73) carried eight of the screened genes. All lineage 3 isolates harbored 8 or 9 internalin genes, except for one isolate that harbored only 7. All lineage 1 isolates harbored *inlF*, while 89.7% of lineage 2 isolates and none of lineage 3 isolates contained *inlF*. In contrast, none of the lineage 1 isolates carried *inlG*, while 66.7% of lineage 2 isolates and 63.6% of lineage 3 isolates harbored *inlG.* All isolates harbored *inlP* except for two lineage 3 isolates (TB0631 and TB0708). *inlF* was absent in all the SL121 (*n* = 2) and SL689 (*n* = 2) isolates in lineage 2 and from isolates in lineage 3. *inlG* was absent in all lineage 1 isolates, as well as SL121, SL375, and SL689 in lineage 2 and SL1965, SL2795, SL2799, and SL2800 in lineage 3.

LIPI-3, encoding listeriolysin S, which is associated with *Listeria* virulence *in vivo* (17, 18), was found in all lineage 1 isolates with the exception of TB0656 from SL59. LIPI-3 was also found in a single lineage 3 isolate, TB0508, which belongs to SL1965. LIPI-4 is composed of a cluster of six genes (*lm4b_02324*, *lm4b_02325*, *lm4b_02326*, *lm4b_02327*, *lm4b_02328*, and *lm4b_02329*) and was identified in 13.7% (10/73) isolates from lineages 1, 2, and 3. In lineage 1, LIPI-4 was found in isolates belonging to SL217 and SL219, in lineage 2, it was found in a single isolate from SL1966, and in lineage 3, it was found in five isolates belonging to SL2350, SL2352, SL2795, SL2799, and SL2800.

### Stress and antimicrobial resistance gene profiles.

**FIG 3:**
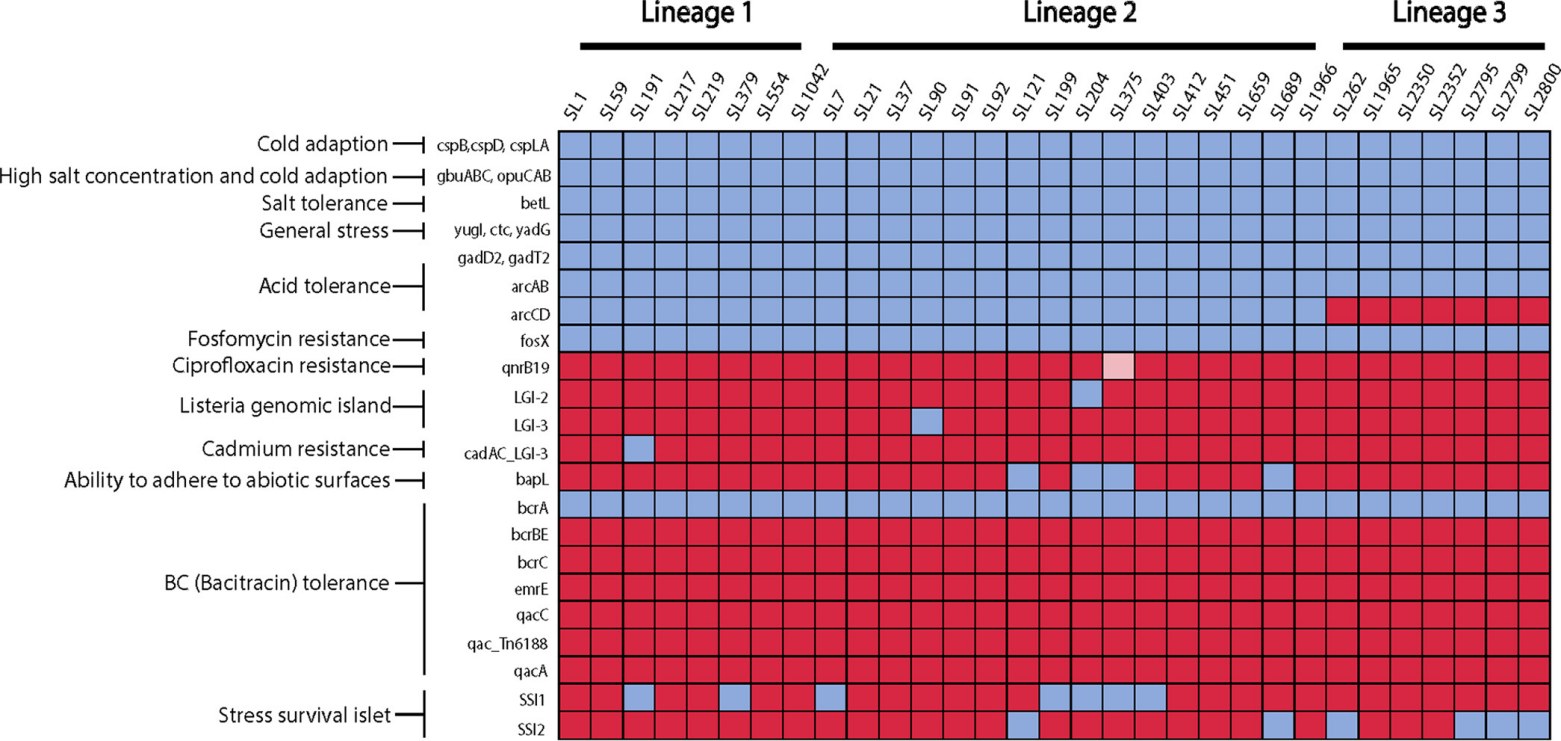
Presence and absence of stress resistance genes grouped by lineage and sublineage. Blue indicates presence, red indicates absence, and pink indicates that the gene was present in at least one of the isolates from a specific SL.

Many of the well-described stress resistance genes were present in all the strains (Fig. 3). All of the isolates (100% [73/73]) contained *csp*, *gbuABC*, *betL*, and *opuCAB*, which contribute to tolerance of cold and osmotic stress. Genes *yugI*, *ctc*, and *ydaG*, encoding general stress proteins, were present in all isolates. Genes encoding proteins involved in the acid tolerance response were variably present. Genes encoding the glutamate decarboxylase acid tolerance system were present in all isolates, while the genes encoding the arginine deiminase system for acid tolerance were not present in all isolates. Notably, *arcA* and *arcB*, encoding arginine deiminase and ornithine carbamoyltransferase, were present in all isolates, but *arcC* and *arcD*, encoding carbamate kinase and arginine/ornithine antiporter, were found only in lineage 1 and lineage 2 isolates and were absent in all lineage 3 isolates.

Stress survival islet 1 (SSI1), associated with growth under low-pH and high-salt conditions (19), was identified in 21.7% of lineage 1 isolates and 51.3% of lineage 2 isolates and was not found in any of the lineage 3 isolates (Fig. 3). Stress survival islet 2 (SSI2), associated with increased tolerance to oxidative and alkaline stress (20), was only identified in SL121 all isolates in lineage 2, one isolate (TB0655) in SL689 lineage 2, and one isolate (TB0453) in SL262 lineage 3. SSI2 was not present in any lineage 1 isolates.

A number of genes known to be associated with tolerance to sanitizers and heavy metals were included in the stress resistance genes that were screened. Of those, benzalkonium chloride tolerance gene *bcrA* was identified in all the isolates. Remarkably, among the isolates collected from ruminants in this research, only isolates of SL204 were found to possess *Listeria* genomic island (LGI)-2, associated with arsenic resistance (21). LGI-3, a recently described *Listeria* genomic island first identified in isolates from food processing facilities (22), was only identified in TB0577 (SL90), an isolate from a fetal infection collected from North Dakota in 2017. Isolates of SL191 were found to have the *cadAC* loci associated with LGI-3 but did not have the other LGI-3 genes present. All isolates were screened for LGI-1, but no isolates had this genomic island.

Antimicrobial resistance genes were screened using the ResFinder database which had 3,077 nucleotide sequences at the time of the analysis. All of the isolates (73/73 [100%]) harbored the *fosX* gene, which confers intrinsic resistance to fosfomycin. Identities were between 88.3% and 100%, and coverage was >99.75%. Only one isolate, TB0689 (L2/SL375), harbored both *fosX* and *qnrB19*, which encodes resistance to quinolones. Genes such as *lin*, *norB*, and *mprF*, which confer resistance to lincosamides, quinolones, and cationic peptides, as well as *tetM* and *tetS*, which confer resistance to tetracycline, were absent in our data set.

### Detection of prophages and plasmids.

**FIG 4:**
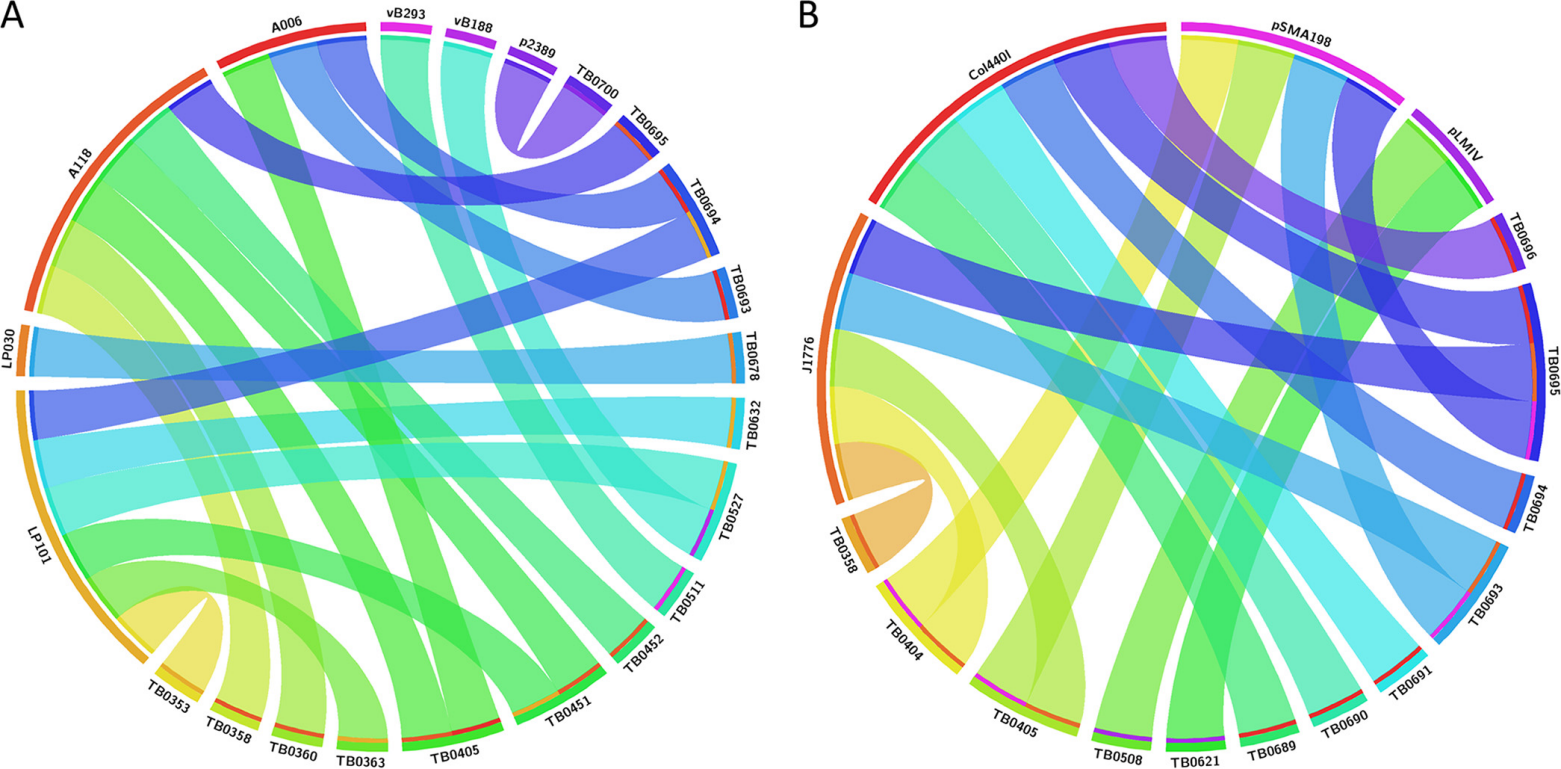
Circos plot showing type of prophage and plasmid sequences found in a set of isolates from ruminant listeriosis cases. (A) Isolates in which prophage sequences were found (20.5% [15/73]); (B) isolates in which plasmid sequences were found (16.4% [12/73]). Isolate IDs are indicated with the suffix TB. The outer colored blocks under the prophage/plasmid names correlate to the color of the inner colored lines in the isolates in which the prophage/plasmid sequence was found.

Prophages were identified using PHASTER and were found in 20.5% (15/73) of the total isolates (Fig. 4A; see also Table S2 in the supplemental material). Prophages were detected in 13.4% (3/23) of the isolates from lineage 1, 28.2% (11/39) of isolates from lineage 2, and 9.1% (1/11) of isolates from lineage 3. Seven different types of prophages were found: 2389 (GenBank accession number NC_003291), A006 (NC_009815), LP-030-3 (NC_024384), A118 (NC_003216), LP-101 (NC_024387), vB LmoS 188 (NC_028871), and vB LmoS 293 (NC_028929). The most common prophage was LP-101 (NC_024387), present in 40.0% (6/15) of the isolates, followed by A118 (NC_003216), present in 33.3% (5/15). All L2/SL191/CC191 strains in this study harbored prophages.

Plasmids were screened using the PlasmidFinder database, which had 460 sequences at the time of the analysis. Plasmid sequences were found in 16.4% (12/73) of the strains. The Col440I plasmid replicon was present in 50% (6/12) of the isolates carrying plasmids, J1776 in 41.6% (5/12), pSMA198 in 33.3% (4/12), and pLMIV in 16.6% (2/12) (Fig. 4B). One isolate, TB0695 (SL191), harbored three plasmids: J1776, pSMA198, and Col440I. Three isolates harbored both J1776 and pSMA198, and eight harbored only one of the plasmid sequences. All of the strains harboring plasmids were isolated from North Dakota (11/12) and Minnesota (1/12) between 2015 and 2020, and the majority belonged to L1/SL191 (4/12) and L2/SL375 (4/12). Interestingly, five isolates from North Dakota (TB0358, TB0405, TB0693, TB0694, and TB0695) harbored at least one prophage (A006, A118, and/or LP101) and one plasmid sequence (J1776, pSMA198, and/or Col440I) at the same time.

## DISCUSSION

### Diversity of L. monocytogenes isolates from ruminant listeriosis.

Previous characterization of ruminant listeriosis isolates from different regions in Europe found that all strains belonged to either phylogenetic lineage 1 or 2, with no lineage 3 isolates identified. Our goal in this study was to assess genomic diversity of L. monocytogenes isolated from ruminant listeriosis cases in the United States, which has not been examined. While the majority of isolates from ruminant listeriosis cases in the Midwest and Upper Great Plains states belonged to lineage 1 and lineage 2, we did find that 15.1% (11/73) of the isolates belonged to lineage 3, which was rarely found in previous studies. In contrast to previous studies, we found that lineage 2 isolates were the most frequently associated with ruminant listeriosis, which accounted for 53.4% of cases, while lineage 1 isolates accounted for 31.5% cases. In a survey of ruminant listeriosis isolates from Italy, Rocha and colleagues found 85% of isolates belonged to lineage 1 and 15% to lineage 2 (23). Dreyer and colleagues found that 63% of ruminant listeriosis isolates from Switzerland and the United Kingdom belonged to lineage 1 and 37% to lineage 2 (11). Papic and colleagues had similar findings, with 70% of ruminant listeriosis isolates from lineage 1 and 30% from lineage 2 (12). None of these studies found isolates from lineage 3, even though the surveys by Dreyer et al. and Papic et al. examined 187 isolates and 164 isolates, respectively, which were larger strain sets than in our study.

An association between clinical manifestations and L. monocytogenes lineages has been identified in multiple studies. Lineage 1 isolates have been associated with neurologic infections, specifically rhombencephalitis, in ruminants (11, 12, 24). In this study, we found that lineage 1 and lineage 3 isolates were associated with neurologic infections. Isolates of SL1/CC1 are most frequently associated with neurologic infections in ruminants (11, 12, 23), which we also observed with 9/9 SL1 isolates from neurologic infections. SL1 isolates are considered hypervirulent in both ruminants and humans (10, 11), and SL1 is one of the most commonly isolated subtypes of L. monocytogenes globally (25). Dreyer et al. found that CC1 and CC4 had a high prevalence in ruminant clinical isolates (81%) (11) and Papic et al. had similar findings, with CC1, CC4-217, and CC412 frequently isolated from clinical cases (12), while in our study, SL1/CC1 and SL219/CC4 represented just 19.1% of the ruminant listeriosis isolates. Hypervirulent L. monocytogenes CC1 has been strongly associated with dairy and meat products, evidence that ruminants are a major reservoir for this subtype, leading to entry into the food supply chain (14). Palacios-Gorba et al. found that the most prevalent SL/CCs collected from ruminant fecal samples on farms were SL1/CC1, SL219/CC4, SL26/CC26, and SL87/CC87 (6). This study found a high prevalence of the hypervirulent clones SL1/CC1 and SL219/CC4, which are responsible for both human infections and ruminant infections, revealing that L. monocytogenes isolates from healthy ruminant fecal samples have some overlap with the isolates from infected ruminants and human clinical infections.

While most studies have focused on assessing the subtypes associated with ruminant neurologic infections, two studies have also investigated subtypes associated with abortions and fetal infections. Papic and colleagues found that while CC1 was most prevalent among ruminant abortion cases (24/128), isolates from CC37 (lineage 2) and CC6 (lineage 1) were significantly associated with abortion cases compared to neurologic cases (12). Similar to the case with our study, Šteingolde and colleagues found that lineage 2 isolates were significantly associated with ruminant abortion cases (26). Among 125 isolates collected over 5 years from ruminant abortion cases in Latvia, only 3 isolates belonged to lineage 1. The isolates were from multiple CCs in lineage 2, and CC29, CC7, CC37, CC14, and CC451 were commonly isolated over the 5 years of the study (26). Many of these subtypes were found in our study, with isolates of SL37/CC37, SL91/CC14, and SL92/CC14 found only in cases of ruminant fetal infection. Isolates of these subtypes were also found to be persistent in the dairy farm environment, with ST14 (CC14), ST37 (CC37), ST91 (CC14), and ST20 (CC20) commonly identified from fecal and soil samples from farms (13), indicating that these subtypes associated with fetal infections are likely circulating on farms.

### Variation in the presence of virulence factors.

Variation in virulence phenotypes is mainly driven by the presence or absence of groups of genes encoding virulence factors (10). While all L. monocytogenes isolates contain the well-described LIPI-1 genes, we noted variation in the presence of internalins, LIPI-3, and LIPI-4. To date, *inlA*, *inlB*, *inlC*, *inlF*, and *inlP* are known to be involved in different stages of infection. *inlA*, *inlB*, and *inlF* are located on the surface of the bacterial cell and interact with surface receptors on the host cells to promote their internalization. On the other hand, *inlC* and *inlP* are secreted in the host cell cytoplasm after bacteria are internalized, facilitating cell-to-cell spread to target organs such as the brain and the placenta (27). In this study, we have confirmed the presence of at least 8 internalin genes in all of the isolates examined (except for one lineage 3 isolate that harbors only 7 internalin genes). Likewise, we identified *inlF* in all lineage 1 isolates, while our findings for lineage 2 are similar to those of Šteingolde et al., who found that *inlF* was absent in all CC/SL121 and CC/SL689 lineage 2 isolates, while all SL91 strains carried *inlF* (26). Thus, the presence of internalins in our data set embodies a high degree of consistency within CC/SL.

As reported previously, LIPI-3 genes were absent in isolates from lineages 2, 3, and 4 (28), with an exception in a fetal infection isolate (TB0508) belonging to lineage 3. LIPI-4, which has been associated with conferring selective tropism for the central nervous system and fetal-placental organs and initially described as exclusive of lineage 1 CC4 (SL219) isolates (10), was identified in SL219 isolates. Additionally, we found that LIPI-4 was present in one fetal infection isolate belonging to lineage 2 and in five novel neurologic infection-associated isolates from lineage 3, indicating a wider presence of this pathogenicity island among genetically diverse isolates.

### Variation in the presence of stress resistance genes.

Stress resistance genes have shown association with specific lineages and CCs as well. The acid tolerance genes *arcC* and *arcD*, important for *in vivo* infection and environmental stress protection, are present in lineage 1 and 2 isolates and are absent in lineage 3 isolates, suggesting that the presence of this gene is lineage dependent, as described previously by Chen and collaborators (29). Furthermore, only one isolate from L2/SL204 harbored arsenic resistance-associated genes, consistent with previous studies by Maury et al. and Gelbivoca et al., in which isolates from CC2, CC14, and CC204 (SL204) have been found to carry this gene, often located on the chromosomal island LGI2 (14, 30). Likewise, SSI2 genes were found only in L2/SL121 as reported previously, where only CC475 and CC121 isolates harbored SSI2 (14, 20).

Previous studies suggest that the presence of plasmids and prophages is variable across phylogenetic subgroups. These mobile elements can carry genes involved in stress survival and antimicrobial resistance (AMR), which poses a great threat due to the horizontal gene transfer. In 2017, Hingston et al. found plasmids in 55% of 166 strains from various STs (31). More recently, 48% of 201 L. monocytogenes strains from ready-to-eat (RTE) foods were found to harbor plasmids (32), and in 2021, Schmitz-Esser et al. assessed 1,921 published genomes from strains representing 14 STs and found that 54% of the strains contained a putative plasmid, with a higher distribution in strains from environmental (48%) and food-related (39%) origins than in those from clinical settings (13%) (33). In this study, we found that 16.4% (12/73) of the ruminant isolates harbored plasmid sequences and 20.5% (15/73) harbored prophages. Our results show that most of the isolates harbored one plasmid sequence (8/12) and/or a prophage (10/15); however, some strains harbored two or more plasmids/prophages or a combination of them, similar to what has been reported previously (31, 34, 35). Screening of putative plasmids is essential to monitor antimicrobial resistance transfer and for implementation of predictive measures to limit the spread of resistance.

Regarding AMR genes, we confirmed that all the isolates included in this study harbored *fosX*, which confers intrinsic resistance to fosfomycin *in vitro* (36). Additionally, only one isolate harbored *qnrB19*, a plasmid-borne quinolone resistance gene associated with reduced susceptibility in enterobacteria (37–39). Although L. monocytogenes rarely develops resistance to antibiotics, some studies have reported an increased presence of AMR genes in isolates from different sources. In Brazil, for example, AMR was detected in around 57% of the isolates recovered from cattle and poultry slaughterhouses, with resistance to sulfonamides the most common feature, along with the presence of *tetC*, *ermB*, and *tetB* (40). Similarly, a study conducted in France that included isolates from food and environmental sources over a 10-year period found isolates resistant to erythromycin, tetracycline, and trimethoprim and detected *ermB*, *tetM*, and *dfrD* among other genes associated with antimicrobial resistance (41). Our findings confirm that the AMR rate in ruminants in the Midwest and the Upper Great Plains is low, and they contribute to the monitoring of the spread of AMR in the region, highlighting the role of L. monocytogenes strains as potential reservoirs of resistance genes that could then be transmitted to humans.

The genetic diversity of isolates from ruminant listeriosis from the Midwest and the Upper Great Plains has not been described before. Although genetic variation within the species is present, L. monocytogenes is still considered highly clonal, sharing around 70% of the gene content among strains. Accessory genes, as well as genetic elements such as AMR genes and plasmids, are responsible for most of the subgroup-specific features and may be the ones involved in the increased frequency of certain CC/SL, playing a key role in the spread of hypervirulent clones within a given population.

### Conclusion.

Our study addressed the gap in data related to genomic characterization of U.S. ruminant listeriosis isolates. We identified a significant number of lineage 3 isolates associated with ruminant listeriosis, which had not been observed in larger isolate sets from cases in other geographic locations (12, 40, 42). With these data, we illustrated the associations between lineage and clinical manifestations in the region, as well as overlaps between frequently isolated sublineages from ruminants and those from human cases. Additionally, we characterized the genetic repertoire of ruminant listeriosis isolates regarding presence of virulence, stress, and AMR genes. Our findings described the molecular epidemiology of ruminant listeriosis cases in the United States and identified L. monocytogenes strains circulating in the area with potential to cause disease in the ruminant population.

## MATERIALS AND METHODS

### Strain collection.

This study was conducted using a total of 73 L. monocytogenes isolates from ruminant listeriosis cases in the Midwest and Upper Great Plains states: North Dakota, South Dakota, Minnesota, Nebraska, and Michigan (Table 1). Isolates were collected from 2015 to 2020. *Listeria* spp. were isolated from tissues submitted to the Veterinary Diagnostic Laboratory at either North Dakota State University (NDSU) or Michigan State University (MSU) for disease investigation. At the NDSU lab, tissue samples were crushed manually in stomacher bags with a 1:10 ratio of sample to brain heart infusion (BHI) broth. *Listeria* agar and broth prepared in-house per package instructions (*Listeria* enrichment base and *Listeria* supplement, Oxford formulation) were inoculated with tissue homogenate and incubated at 37°C in the ambient atmosphere for 16 to 24 h. *Listeria* agar was observed for dark/black colonies; if no suspicious colonies were present on *Listeria* agar, the broth was incubated for an additional 24 h before inoculation to Trypticase soy agar (TSA) with 5% sheep’s blood and *Listeria* agar. TSA with 5% sheep’s blood was incubated at 35°C in 5% CO_2_; *Listeria* agar plates were incubated at 37°C in the ambient atmosphere. After 18 to 24 h of incubation, plates were observed for typical small, white, beta-hemolytic colonies on blood agar and dark/black colonies on *Listeria* agar. At the MSU lab, tissue samples were macerated and plated directly onto a colistin-nalidixic acid with 5% sheep blood agar and modified Oxford medium agar plates (MOX) (Hardy Diagnostics, Santa Maria, CA). All samples were also enriched for *Listeria* using University of Vermont enrichment medium (UVM; Hardy Diagnostics, Santa Maria, CA). In brief, 25 mg of the macerated tissue was added to 225 mL of UVM broth and incubated overnight at 35 to 37°C in a 5% CO_2_ incubator. After the overnight incubation, the broth was plated onto a MOX plate using a sterile cotton swab. Simultaneously, 100 μL of the broth was also added to 10 mL of fresh UVM broth for a secondary enrichment. After overnight incubation of the secondary enrichment, the broth was plated onto MOX agar as described above. All plates were incubated at 35 to 37°C in a 5% CO_2_ incubator and observed for growth of *Listeria* spp. At both the NDSU and MSU labs, any suspect colonies were streaked onto a 5% sheep blood agar plate, incubated overnight as described above, and identified using matrix-assisted laser desorption ionization–time of flight mass spectrometry (MALDI-TOF MS) (Bruker Microflex LT; Bruker Daltonics, Bremen, Germany).

Isolates identified as L. monocytogenes were then classified into three clinical manifestation categories based on their source of isolation and diagnosis. The categories were as follows: (i) neurologic, for isolates obtained from brain or brain stem tissues; (ii) fetal infection, for isolates obtained from placental or fetal tissues; and (iii) other, for isolates obtained from tissues such as intestine or lung.

### Whole-genome sequencing.

Isolates were stored at −80°C in BHI broth with 15% glycerol and inoculated into BHI broth and incubated at 37°C for 24 h prior to use for DNA extraction. Genomic DNA was extracted using either the Qiagen DNeasy blood and tissue kit (Qiagen, Valencia, CA) or a modified phenol-chloroform protocol (43). Quantity of the extracted DNA was assessed using the Quant-iT Pico green double-stranded DNA (dsDNA) assay kit (Thermo Fisher Scientific) and a Qubit fluorometer (Thermo Fisher Scientific), in addition to a NanoDrop spectrophotometer (Thermo Fisher Scientific, Carlsbad, CA). The Nextera XT DNA sample preparation kit (Illumina, San Diego, CA) was used for DNA library preparation. Paired-end whole-genome sequencing (2 × 250 bp) was performed on the Illumina MiSeq system. Sequencing depth ranged from 35× to 70×. For a subset of 24 strains, short-read sequencing was complemented with long-read sequencing using a MinION flow cell (Oxford Nanopore Technologies, Oxford, UK). For this set of strains, the modified phenol-chloroform method was used to extract DNA, which was used with the rapid barcoding kit (Oxford Nanopore Technologies) to prepare libraries for sequencing. Sequencing was performed on the MinION flow cell.

Quality control of the reads was performed using FastQ (44), and reads with quality values below Phred 20 were excluded from the analysis. Raw reads were processed to remove low-quality bases and adapter sequences using Trimmomatic v. 0.39 (45). For the 49 genomes with only short reads, *de novo* assembly was performed using SPAdes v. 3.15.2 (46) with the default settings. Assembly of the 24 genomes with short and long reads was conducted with Unicycler (https://github.com/rrwick/Unicycler). The assemblies generated by SPAdes or Unicycler were annotated using PROKKA v. 1.14.6 (47) with the default parameters using the L. monocytogenes 10403S genome as a reference. To classify isolates into genetic lineages, a reference tree based on cgMLST was generated using RaxML (48). The genome of L. monocytogenes lineage 4 strain FSL J1-208 (GenBank accession number NZ_CM001469.1) was included as an outgroup to root the phylogenetic tree. We then included 10403S (NC_017544.1) as a reference for the most common lineage 2 CC (CC7) and the ScottA (NZ_CP023862.1) strain as a reference for the most common lineage 1 CC (CC1). Sequences were downloaded from GenBank (NCBI) and included in the analyses as controls. The resulting maximum-parsimony tree (based on the consensus of 100 trees) clearly segregated the four lineages. The resulting tree was visualized and edited using iTOL v. 5 (49).

### Genome analyses.

Assembled genome files were submitted to the Bacterial Isolate Genome Sequence database of L. monocytogenes (BIGSdb-Lm) (https://bigsdb.pasteur.fr/listeria/) for classification into sublineages (SLs) and cgMSLT types (CTs). GrapeTree was used to create a minimum spanning tree based on the cgMLST profiles (50). Annotated assemblies generated in Prokka were used to calculate the core and accessory genome with Roary, where genes present in 95% of the genomes with an identity of at least 95% were assigned as core genes (51). The identification of prophages in the genomes was conducted with PHASTER (52), where “intact” regions with sequence lengths of >20 kbp were used for the assessment. Identification of plasmids was conducted using ABRicate v. 0.8.10 (https://github.com/tseemann/abricate) with the predownloaded PlasmidFinder database (53, 54) and were visualized with Circos (55). Assembled genomes were used as input for the screening of virulence and resistance genes using ABRicate v. 0.8.10 (https://github.com/tseemann/abricate). Virulence genes were identified by comparison against the Virulence Factor Database (VFDB) (56). Antimicrobial resistance genes were identified by using Resfinder (57) (accessed on 7 April 2022). An in-house database was used to screen for additional virulence factors using ABRicate v. 0.8.10, with the minimum identity and coverage cutoffs values set by default. This database included a total of 148 sequences of stress tolerance genes and virulence factors, such as internalin and pathogenicity islands, retrieved from the *Listeria* database hosted by the Pasteur Institute, Paris, France (Table S1).

### Statistical analyses.

Chi-square tests were used to analyze the association between lineage and clinical manifestation and implemented in R v. 4.1.2. This allowed us to determine if a particular type of clinical manifestation was significantly associated with a specific lineage or if lineages were significantly associated with a clinical manifestation. A *P* value of <0.05 was considered to be statistically significant. The plot was made using the ggplot2 package in R v. 4.1.2.

### Data availability.

All sequence data are available in the Sequence Read Archive at NCBI. SRR accession numbers are provided in Table 1.
