# Supplementary material for: Comparative Genomics of Listeria monocytogenes Isolates from Ruminant Listeriosis Cases in the Midwest United States
Source: Microbiol Spectr. 2022 Oct 31;10(6):e01579-22. doi: 10.1128/spectrum.01579-22 (PMC9769944; doi:10.1128/spectrum.01579-22)
Supplement: Supplemental file 1 — Supplemental material. Download spectrum.01579-22-s0001.pdf, PDF file, 0.1 MB [file spectrum.01579-22-s0001.pdf]

Supplemental Table 2. Phages in *L. monocytogenes* isolates used in this study.

ID	Lineage	SL	Clinical manifestation	Prophage
TB0405	1	191	Neurologic	A006 [NC_009815]; LP-030-3 [NC_024384]
TB0693	1	191	Fetal infection	A006 [NC_009815]
TB0695	1	191	Neurologic	A118 [NC_003216]
TB0358	2	7	Neurologic	A118 [NC_003216]
TB0360	2	7	Neurologic	A118 [NC_003216]
TB0452	2	7	Other	A118 [NC_003216]
TB0451	2	7	Neurologic	LP-101 [NC_024387]; A118 [NC_003216]
TB0363	2	21	Neurologic	LP-101 [NC_024387]
TB0632	2	37	Fetal infection	LP-101 [NC_024387]
TB0353	2	91	Fetal infection	LP-101 [NC_024387]
TB0527	2	92	Fetal infection	LP-101 [NC_024387]; vB LmoS 188 [NC_028871]
TB0511	2	121	Fetal infection	vB LmoS 293 [NC_028929]
TB0694	2	659	Other	LP-101 [NC_024387]; A006 [NC_009815]
TB0678	2	689	Other	LP-030-3 [NC_024384]
TB0700	3	2795	Neurologic	2389 [NC_003291]
